# Chronic intracranial subdural hematoma after spinal anesthesia for a cesarean section: a case report

**DOI:** 10.1186/s13256-021-03100-0

**Published:** 2021-10-07

**Authors:** Delayehu Bekele, Mehari Bayable, Alemayehu Bedane

**Affiliations:** 1 Department of Obstetrics and Gynecology, Saint Paul’s Hospital Millennium Medical College, P.O.Box 143079, Addis Ababa, Ethiopia; 2 Department of Obstetrics and Gynecology, Saint Paul’s Hospital Millennium Medical College, P.O.Box 1271, Addis Ababa, Ethiopia; 3 Department of Radiology, Saint Paul’s Hospital Millennium Medical College, P.O.Box - 143079, Addis Ababa, Ethiopia

**Keywords:** Spinal anesthesia, Complications, Subdural hematoma, Post-dural puncture headache

## Abstract

**Background:**

Subdural hematoma is a rare, potentially devastating, yet curable complication of spinal anesthesia. Differentiation between post-dural puncture headache and subdural hematoma can be difficult, resulting in a delay in diagnosis.

**Case presentation:**

We present a 28-year-old Ethiopian female patient who underwent elective cesarean section under spinal anesthesia and returned to the emergency department after 1 month with a worsening headache. Brain computed tomography revealed a chronic subdural hematoma with a significant midline shift. The patient recovered completely after surgical evacuation.

**Conclusions:**

A high index of suspicion and close attention to the pattern and characteristics of the headache, coupled with a meticulous neurologic examination and neuroimaging, can help to achieve timely diagnosis of this serious entity. Investigation with head computed tomography or magnetic resonance imaging is vital.

## Background

Spinal anesthesia is a safe and effective alternative to general anesthesia for cesarean delivery. Post-dural puncture headache (PDPH) is one of the common complications of spinal anesthesia that is usually managed with bed rest in a flat position, hydration, and simple analgesics [1]. PDPH is usually a benign self-limited condition, but it may also be a sign of a rarer but more serious complication like intracranial hemorrhage [2, 3]. Patients with complaints of PDPH often seek treatment and usually respond to conservative measures like bed rest, oral analgesics, and hydration. If the PDPH is persistent and/or is having associated neurologic findings, the patient should be investigated for an intracranial hemorrhage. Subdural hematoma after neuraxial anesthesia or dural puncture is a relatively rare complication, with around 50 cases being reported in the literature [4, 5]. Here, we present a case of a chronic subdural hematoma (CSDH) diagnosed 31 days after spinal anesthesia for cesarean delivery.

## Case presentation

A 28-year-old Ethiopian pregnant (G2, P1) woman presented to our emergency obstetrics department with a complaint of a worsening headache and easy fatigability for 3 days. It was a continuous vague type of global headache that became worse over 24 hours and was associated with easy fatigability, generalized malaise, and nausea. She had undergone an elective cesarean section at our hospital 31 days before her current presentation with an indication of “previous cesarean scar and big baby.” She had a normal pregnancy with an uneventful antenatal follow-up. She had no history of any known chronic medical illness like hypertension, diabetes, cardiac or renal disease, coagulopathy, or any bleeding tendency. She has no history of primary headache, migraine, or any trauma.

For her cesarean delivery, spinal anesthesia was given in a sitting position with a 22-gauge Quincke spinal needle at the L3–L4 interspace using 12.5 mg of 0.5% isobaric bupivacaine. The puncture was successful on the first attempt, and the course of anesthesia was without incident. Her intraoperative vital signs were stable, and the surgery was completed uneventfully. She had a smooth postoperative course and was discharged on the third day. She had no headache at the time of discharge. She was in a relative resting state being cared for by her family and did not return to work. She had no history of trauma or fall accident. About 8 days later, she developed a mild global headache for which she was managing herself with rest and paracetamol *per os* as needed. The headache increased gradually and became associated with nausea and easy fatigability.

Upon presentation, on the 31st postoperative day, she complained of severe global headache with worsening over 3 days that was not position dependent and was unrelieved with rest and analgesics. On physical examination, there was no vital sign derangement. She was fully conscious and oriented with no mental status change. All meningeal signs were negative, and her motor function was intact bilaterally.

**Fig. 1:**
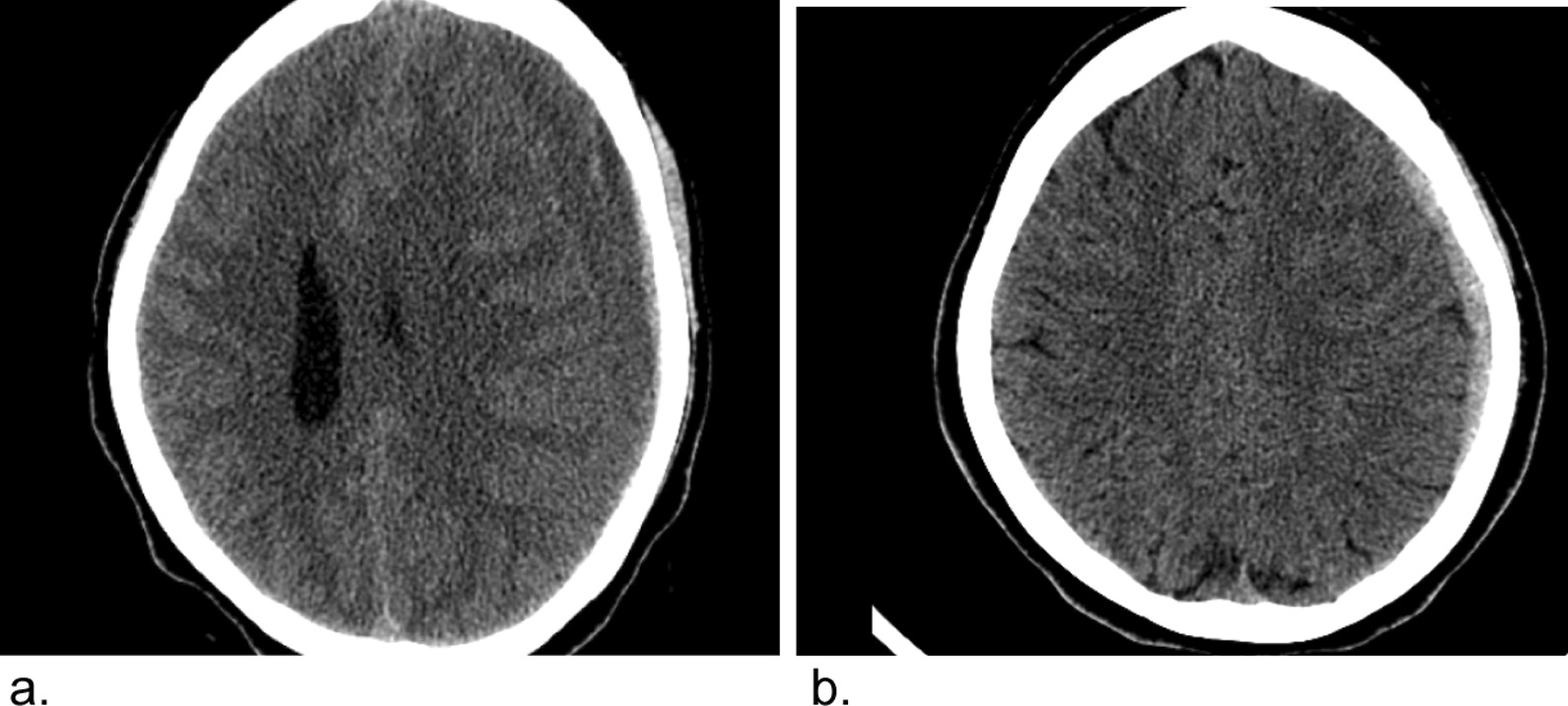
Pre- and postoperation Axial precontrast CT scan: a large left frontoparietal chronic subdural hematoma with areas of acute bleeds (**a**) that shows significant improvement after operation (**b**)

Laboratory data revealed normal complete blood count, prothrombin time (PT), partial prothrombin time (PTT), and international normalized ratio (INR). CT scan of the head revealed a layered hypodense crescent-shaped extra-axial left frontoparietal collection with areas of linear and crescentic hyperdensities (Hounsfield unit of 50). There was a mass effect on the underlying brain resulting in a midline shift of 13 mm. The width of the lesion measured 15 mm at the level of the bodies of the lateral ventricles. The finding was consistent with chronic subdural hematoma with areas of acute bleeds (Fig. 1a).

After emergent consultation with a neurosurgeon, the patient was admitted to the neurosurgical ward, and hematoma evacuation was achieved using burr-hole placement. She had a smooth immediate postoperative course and was discharged home 3 days later. Follow-up CT showed a crescentic left frontoparietal hyperdense extra-axial collection with a width of 3.8 mm and midline shift of 2.2 mm at the level of the bodies of the lateral ventricle (Fig. 1b), a finding suggestive of a largely improved left frontoparietal subdural hematoma. Clinically, she had an uneventful recovery with full resolution of her symptoms. On subsequent follow-up at 6 months, the patient had no residual symptoms or neurologic deficit.

## Discussion and conclusions

We presented a case of subdural hematoma that occurred more than 1 month after spinal anesthesia lumbar puncture was done for cesarean section. The case demonstrates the importance of having a high index of suspicion and the need for timely investigation with imaging in patients with suggestive symptoms.

Spinal anesthesia is a commonly used method to achieve anesthesia in obstetric patients [6, 7]. It has the advantage of avoidance of complications of general anesthesia and a more direct experience of childbirth. But it has complications such as hypotension, post-dural puncture headache, nerve damage, meningitis, intracerebral hemorrhage, spinal, and cranial hematoma. Intracranial subdural hematoma is a rare but potentially lethal complication that can occur after neuraxial anesthesia or myelography. An incidence of 1 in 500,000 obstetric procedures has been estimated for intracranial SDH following spinal anesthesia, but most authors believe that the true incidence of SDH after dural puncture might be higher as many cases go unreported [8].

The exact mechanism for PDPH is not known, but continued cerebrospinal fluid (CSF) leakage, leading to a reduction in CSF pressure, is often blamed [1, 4, 9]. Predisposing factors for PDPH and subsequent development of subdural hematoma include pregnancy, dehydration, multiple dural punctures, using large gauge needles, coagulopathy, cerebral vascular abnormalities, brain atrophy, and alcohol consumption [4]. In reviewing 35 cases of intracranial hemorrhage following spinal anesthesia, Amorim *et al.* found that there were no apparent predisposing factors in 15 of these patients. Among those who did have such risk factors, the most common were pregnancy, multiple punctures, use of anticoagulants, intracranial vascular abnormalities, and brain atrophy [10]. The contributing factors in our patient could be the pregnancy and the large bore needle used for the puncture.

Severe headache after spinal anesthesia in a pregnant patient has a broad differential diagnosis, including post-dural puncture headache and intracranial pathologies, so a high index of suspicion and early investigation for patients having a persistent headache is lifesaving. It is not easy to differentiate between the neurological symptoms of intracranial hypotension of PDPH and SDH. In the SDH cases presented by Amorim, in addition to headache as the chief complaint, 89% of the patients presented with at least one of the following signs: vomiting, diplopia, cognitive changes, or altered mental status, or focal neurologic signs [10]. Also, the change in the headache characteristics from postural to nonpostural has been mentioned as a warning sign that intracranial hemorrhage may be complicating simple intracranial hypotension. Our case illustrates the importance of early and careful assessment of post-spinal headache as it could be more serious than just benign PDPH. The International Headache Society has developed a list of criteria to help differentiate PDPH from other, more serious complications of dural puncture [11]. Accordingly, in PDPH, the pain worsens or develops within 15 minutes of assuming an upright position and improves within a similar period after the individual lies down, and it develops within 5 days after the puncture and disappears spontaneously within 1 week, or up to 48 hours after an epidural blood patch is employed. If the clinical presentation is apart from these criteria, one should consider the more severe possibilities and proceed to imaging modalities like CT scan, MRI, and angiography. On a head CT, acute SDH is readily visualized on as a high-density crescentic collection across the hemispheric convexity, while subacute and chronic SDH appear as isodense and hypodense crescent-shaped lesions, respectively. Brain MRI is more sensitive than head CT for the detection of intracranial hemorrhage. MRI has also the additional advantage for the detection of small SDH and tentorial and interhemispheric SDH. Noninvasive angiography (such as MRA or CTA) or conventional cerebral angiography may be indicated for evaluation of SDH, particularly when there is no history of trauma and no obvious cause. In our patient, headache was nonpostural and persistent for more than a month, which mandates imaging.

As with any other SDH, the management is either conservative or surgical. Various treatments for chronic SDH after dural puncture have been reported, including observation, surgical resection of the hematoma, epidural blood patch, or both surgical resection and epidural blood patch [12–15]. The management is decided based on the patient’s neurological symptoms and the size of the hematoma. Small hematomas often resolve spontaneously, and therefore the risk of surgical evacuation is not justified. Early blood patching after the presentation of PDPH may decrease the risk of subdural bleeding through the prevention of CSF loss [16]. Surgical evacuation of the clot is indicated in cases of larger hematomas. In the Amorim study, 27 of the 35 cases required surgical drainage [10], and in the Zeidan series, surgery was performed in 20 of 25 patients [4]. In our patient, severe headache refractory for conservative management, the size of the mass, and significant cerebral shifting on brain imaging were clear indications for surgical intervention.

In conclusion, SDH is a rare, serious yet curable complication of spinal anesthesia, and pregnant women seem to be more susceptible to the development of SDH after spinal anesthesia. Differentiation between PDPH and SDH is difficult, and a high index of suspicion is crucial for timely diagnosis. All patients developing PDPH not relieved by conservative measures, as well as the change of PDPH from postural to nonpostural, require careful assessment and follow-up for early diagnosis and management of possible SDH. Such patients should be investigated with a head CT or MRI.

## Data Availability

Not applicable

## Abbreviations

CSF: Cerebrospinal fluid
CT: Computed tomography
CTA: Computed tomography angiography
INR: International normalized ratio
MRA: Magnetic resonance angiography
MRI: Magnetic resonance imaging
PDPH: Post-dural puncture headache
PT: Prothrombin time
PTT: Partial prothrombin time
SDH: Subdural hematoma
